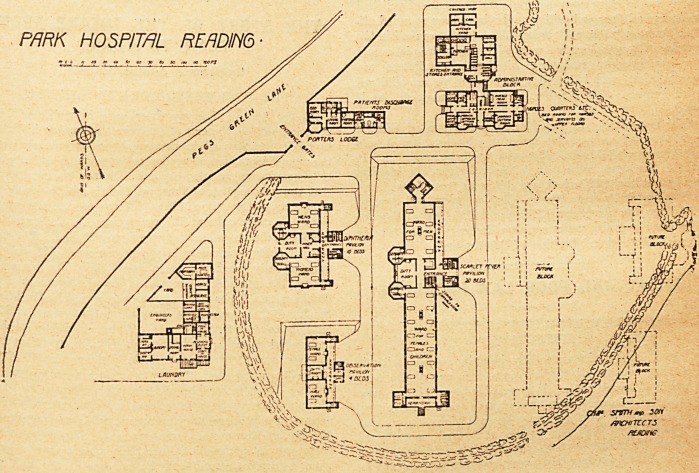# The Park Hospital for Infectious Diseases, Reading

**Published:** 1907-01-05

**Authors:** 


### THE PARK HOSPITAL FOR INFECTIOUS DISEASES, READING.

SOME time since a site of ten acres, situate to the north-west of Prospect Park, was obtained for building purposes, and the hospital was practically completed by the end of 1905, and was opened during the summer of 1906. Being only two miles west of the Reading Town Hall, it is within easy distance of the districts from which its patients will chiefly be drawn. The site is a fine one, sloping towards the south, and it lies over the lower eocene formation. It is entered from Pegs Green Lane at the western end of Tilehurst Road.

On passing the gates, the porter's lodge, containing four rooms, is seen on the left hand. Attached to the lodge, but having a separate entrance, is the discharging block, which is fitted up with a bath, and has dressing-rooms and a waiting-room.

To the east is the administrative block, which includes the kitchen department and the residences for the staff. The kitchen is fitted up with Barford and Perkins apparatus for cooking by steam, and there are also coal and gas ranges.

The residential part of the block is three stories high. On the ground floor are the medical officers' rooms, matron's rooms, nurses' mess-rooms and sitting-rooms, dispensary, etc. The first floor is chiefly given up to sleeping accommodation for the nursing staff, and the second floor for the domestics. The whole of this department, as also the laundry, is large enough for a much higher number of patients than can be taken in at present; but when further room is wanted it will only be necessary to build the blocks for the additional number of beds, and, of course, this is the principle on which all such hospitals should be erected. It may at first make the cost per bed seem rather high; but, in the end, it saves money.

The hospital proper consists of three blocks: one for scarlet fever, one for diphtheria, and one for observation of doubtful cases. All these blocks are placed on the western side of the main central road, and space is thus left for three similar symmetrically-placed pavilions on the eastern side. The pavilions are arranged with their long axes running north-north-east and south-south-west, and this arrangement ensures a large amount of sunshine for the wards.

The scarlet fever pavilion is by far the largest of the three. It is divided into two unequal parts by the entrance hall and the nurses' duty-room; and over this part of the block is a convalescent-room. To the south is the ward for women and children. It contains sixteen beds. At the end are the closets and baths, and between the projections for these is a verandah. Between the ward and the nurses' duty-room is a single-bedded ward which runs out considerably beyond the line of the wards, and is thus provided with cross-ventilation. The male ward is to the north. It contains eight beds, and has a single-bedded ward similar to the one on the female side. The sanitary annexe projects from the extreme end of this ward; and we like this arrangement better than the one on the female side; but in both cases there are cross-ventilated passages. A verandah runs along the eastern side of the male ward, and it is deserving of notice that the glass roof of this verandah is placed lower than the top section of the windows, by which arrangement the efficient cross-ventilation of the ward is not interfered with.

The diphtheria block runs parallel with the scarlet fever one, but is at a sufficient distance from it. In plan it is similar save that it contains only ten beds, having two wards of four beds each and two single-bedded wards, the latter being arranged on the same sensible plan as those in the scarlet fever block. There is a verandah running along the whole of the eastern aspect of this pavilion, and opening off the ends of this are the sanitary annexes.

The observation or isolation block is in line with the diphtheria one. It contains two wards with two beds each, and all the arrangements are quite good.

The laundry block lies at the western side of the site. It is arranged in the form of a square, one-fourth of which is given up to the engineer's yard, and the various parts of the laundry are conveniently placed around two sides of the yard. It is fitted up with Barford and Perkins admirable machinery, and there is a modified Washington Lyons disinfector. Attached to this block are the mortuary, post-mortem-room, and the ambulance-house.

Throughout the pavilions each bed has two thousand cubic feet of air space and one hundred and forty-four superficial feet of floor space. The internal walls are covered with impervious material; the floors are of polished teak, and all floor and ceiling angles have been rounded off. The Taunton "Diagnal" bedsteads and cots are used in the wards. The pavilions are warmed by open fire-places and by low pressure radiators. Shorland's ventilators are placed behind each bedstead, and the windows are all fitted with hoppers. For lighting purposes incandescent gas is used.

The cost of the site was £2,325, and a sum of £20,000 was obtained on loan for building expenses, but it was considered probable that this sum would be exceeded. It would, therefore, seem that, without the site, the hospital has cost at least £500 a bed, and this is far from being cheap.

The architects were Messrs. Charles Smith and Son, of Reading; the contractors, Messrs. Collier and Catley, of Reading; and the engineers were the well-known firm of Messrs. Barford and Perkins, of Peterborough.

## Figures and Tables

**Figure f1:**